# Identification of process parameters influencing product quality in mammalian cell culture

**DOI:** 10.1186/1753-6561-9-S9-O4

**Published:** 2015-12-14

**Authors:** Albert J Paul, Friedemann Hesse

**Affiliations:** 1 Institute of Applied Biotechnology (IAB), Biberach University of Applied Sciences, 88400 Biberach, Germany

## Background

Keywords: Product quality, mAbs, mammalian cell culture, CHO cells, protein aggregation

Monoclonal antibodies (mAbs) are successful biotherapeutics in the treatment of various diseases [1]. During manufacturing of mAbs higher molecular weight (HMW) aggregates can be formed during upstream (USP) and downstream (DSP) processing, which negatively influence product yields, reduce the therapeutic efficacy of the mAbs and trigger immunogenic responses upon administration[2,3]. Reducing the level of aggregates during USP could improve the production of biopharmaceuticals and reduce the burden on expensive DSP removal of the HMW species[4]. However, the lack of analytical tools to detect mAb aggregates in USP restricts understanding the origin of the aggregates and identifying cell culture conditions influencing product quality to reduce the level of mAb aggregates[5]. We present a high-throughput compatible method which allows quantification of mAb aggregate formation directly in cell culture samples of Chinese hamster ovary (CHO) cells replacing falsifying, laborious and time-consuming chromatographic methods. Using this new methodology, we have screened for different culture conditions effecting mAb aggregate formation in a non-producing and a mAb producing CHO cell line. Finally, we have identified important process parameters to influencing protein aggregation in mammalian cell culture. Hence, our work demonstrates that the formation of mAb aggregates can be assessed directly in mammalian cell culture and product quality can be controlled by the selection of certain cell culture process parameters.

## Material and methods

MAb aggregation of two different CHO DG44 cell lines was investigated using different analytical methods. LDH level and mAb concentration were determined using a Konelab™ 20XT analyzer (Thermo Scientific). Cell concentration was assessed using NyONE cell imager (SynenTec). To distinguish between the different mAb aggregates formed in cell culture, aggregate controls were induced using high salt concentration (0.5 M NaCl). The induced aggregates were used to identify extrinsic fluorescence dye concentration and instrument settings for high throughput analysis of bioprocess samples. Formation of soluble mAb aggregates was assessed using the previously described fluorescence dye-based aggregation (FDBA) assay [5]. The formation of larger particles was measured with 10 µM 4-4-bis-1-phenylamino-8-naphthalene sulfonate (Bis-ANS) using fluorescence microscopy on a NyONE cell imager. In order to compare the cell culture samples, the particle count was normalized to the lactate dehydrogenase (LDH) activity in the supernatant of the CHO cells. We screened for cell culture parameters influencing mAb aggregate formation using a statistical experimental design with a linear model (fractional factorial design, resolution IV)by varying initial pH-value (6.8-7.6), osmolality (333-533 mOsm/kg), agitation (100-160 rpm) and culture additives such as the productivity enhancer valproic acid (VPA, 0-4 mM) and antifoam (0-0.04%).

## Results

**Figure 1:**
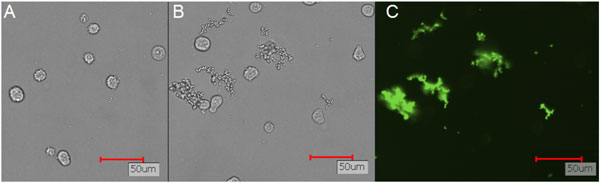
**Fluorescence microscopy images of mAb aggregates in presence of CHO cells**. (A) Transmitted light images of CHO DG44 cells, (B) NaCl-induced mAb aggregates in presence of CHO DG44 cells and (C) Bis-ANS labeled mAb aggregates in the presence of CHO DG44 cells.

Exposure to high salt concentration led to the formation of large mAb aggregates, which were clearly distinguishable from CHO cells due to size and morphoplogy (Figure 1, A+B). After fluorescence labeling of the mAb aggregates with 10 µM Bis-ANS, only mAb aggregates were visible using green emission upon UV excitation (Figure 1, C).

The NyONE sofware enabled further characterization of the mAb aggregates regarding size and number (Nuclei count, NC). Analysis of cell culture samples over time revealed an increase of these fluorescent particles, indicating large particle formation during mAb production in CHO cells. We used this methodology to screen for cell culture parameters influencing mAb aggregate formation in a non-producing (Mock) and a producing (Producer) CHO cell line according to a design of experiment (DoE) procedure (Table 1).

**Table 1:** Cell culture parameters influencing cell growth, mAb productivity and protein aggregation.

	Cell concentration [Cells/mL]		mAb productivity [pg/Cell × day]		FDBA assay [RFU]		Particle formation [NC/U LDH]	
*Parameters*	*Mock*	*Producer*	*Mock*	*Producer*	*Mock*	*Producer*	*Mock*	*Producer*
pH value	0	0	n.a.	0	0	0	0	0
Osmolality	-	-	n.a.	+	0	0	-	-
Agitation	0	0	n.a.	0	+	+	+	+
VPA	0	-	n.a.	+	0	0	0	+
Antifoam	0	0	n.a.	0	0	0	0	0

Result of the DoE screening: (+) Positive effect on response variable, (-) negative effect on response variable, (0) no effect on response variable, (n.a.) not analyzed.

The cell growth of both cell lines was negatively influenced by an increase of osmolality. Furthermore, VPA negatively influenced final cell concentrations of the mAb producing CHO cell line. Besides VPA, osmolality enhanced specific mAb productivity. Only agitation increased the fluorescence in the supernatant, indicating the presence of soluble aggregates, whereas osmolality reduced the level of large particles in both cell lines. This negative correlation of osmolality is surprising, since an osmolality increase e.g. by high salt concentration can lead to mAb aggregate formation. The slight increase of osmolality could have an impact on the cell rather than to the product resulting in a higher specific productivity and less formation of larger host cell or mAb aggregate particles. VPA only increased the particle formation of the mAb-producing CHO cell line, indicating that VPA addition forms product-related aggregates rather than host cell particles. Furthermore, it was shown that VPA also altered the N-glycosylation profile of the mAb. Finally, we could show that the negative impact of VPA on mAb aggregation was concentration-dependent and that the VPA-induced mAb aggregates were formed intracellular and not by the presence of the fatty acid in the supernatant [6].

## Conclusions

We show that protein aggregation of mAbs can be assessed directly in cell culture samples without falsifying pre-purification procedures. Using our developed methods we have identified critical parameters influencing protein aggregation in mammalian cell culture. Moreover, we show that mAb aggregation occurs on two levels during USP: (1) the cellular level and (2) the bioprocess level. Our results indicate how much protein can be lost due to upstream protein aggregation and help to improve production of biopharmaceuticals by reducing the burden on expensive DSP.

## Acknowledgements

This research was supported by the German Federal Ministry of Education and Research (Grant No. 0315342A). The authors are grateful to Rentschler Biotechnologie GmbH for providing the mAb-producing CHO cell line.
